# Multi-Spectroscopic Approach for the Non-invasive Characterization of Paintings on Metal Surfaces

**DOI:** 10.3389/fchem.2020.00289

**Published:** 2020-04-16

**Authors:** Monica Albini, Stefano Ridolfi, Chiara Giuliani, Marianna Pascucci, Maria Paola Staccioli, Cristina Riccucci

**Affiliations:** ^1^Institute for the Study of Nanostructured Materials, National Research Council, Rome, Italy; ^2^Ars Mensurae, Rome, Italy

**Keywords:** painting, metal, spectroscopy, FTIR, SEM-EDS, MA-XRF, imaging

## Abstract

The aim of this study is to propose a non-invasive multi-spectroscopic approach for the characterization of oil painting artworks that use a copper plates as substrate in place of a canvas. Indeed, in the last decade, many studies have been conducted on artworks made of single materials (e.g., paintings, stones, metals). However, the characterization and conservation of composite artifacts has never be fully investigated even though many masterpieces were created using this particular technique. In this work, several spectroscopic techniques such as Infrared Spectroscopy (FTIR), Energy-Dispersive X-Ray Fluorescence spectroscopy (EDXRF), and high spatial resolution Field Emission Scanning Electron Microscopy coupled with Energy Dispersive X-ray Spectroscopy (EDS), and Optical Microscopy (OM) were performed. The obtained results allowed to fully characterize the micro-chemical and microstructural features of the painted surfaces and of the metal plate. Particularly effective was the use of MA-XRF, resulting in the chemical map of the painted surfaces. Furthermore, traces of the mechanical preparation of the plate were found under the painted layers. Finally, the interface area between the paint film and the metallic support was characterized at a micro scale. This was particularly important in order to identify the degradation products formed by the interaction between the fatty acids of the binder and copper-based substrates.

## Introduction

The technique of oil painting on a metal plate is not renowned by the public and the full potential of these artworks is not yet exploited. The practice of painting on metallic surfaces, in particular iron and copper, originated in Europe. An extraordinary example of this technique is the oil painting on copper supports, that started in the 16th century and had a huge growth in Europe during the 17th and 18th century (Graaf, 1972; Horovitz, 1986, 2017; Komanecky et al., 1998; Wadum, 2017). Magnificent artists like Rembrandt, Guido Reni, Diego Velasquez, and Peter Paul Rubens among others, created masterpieces using such a peculiar technique. More than two-thousand extant paintings on metals are exhibited in European and American museums, excluding the far larger production of portraits miniatures on copper (Komanecky, 1990). Indeed, the Galleria degli Uffizi in Florence has, in its collection, more than 500 portrait miniatures using a copper plate as support. The conservation of these artifacts is, thus, extremely important. Nevertheless, few data are available about this painting technique and about the conservation methodology to adopt to preserve them (Thistlewood and Northover, 2009; Pitarch et al., 2014; Veiga et al., 2014, 2015; Fuster-Lopez and Mecklenburg Marion, 2017; Colantonio et al., 2018).

The interesting challenge about these incredible artifacts is their composite nature. In fact, conservation activities have to deal with multiple materials and different degradation processes related to the metallic substrate, the organic paint film, and their interface interactions (Catalano et al., 2019; Keune et al., 2019; Rosi et al., 2019). Furthermore, an additional obstacle to the characterization of these objects is the impossibility to analyze the stratigraphy of the painting. In fact, because of their artistic value, cross-section destructive analyses are precluded on these artifacts. Thus, it is important to develop a systematic approach that exploit non-invasive analytical techniques for the study and characterization of oil paint on a metal substrate.

**Figure 1:**
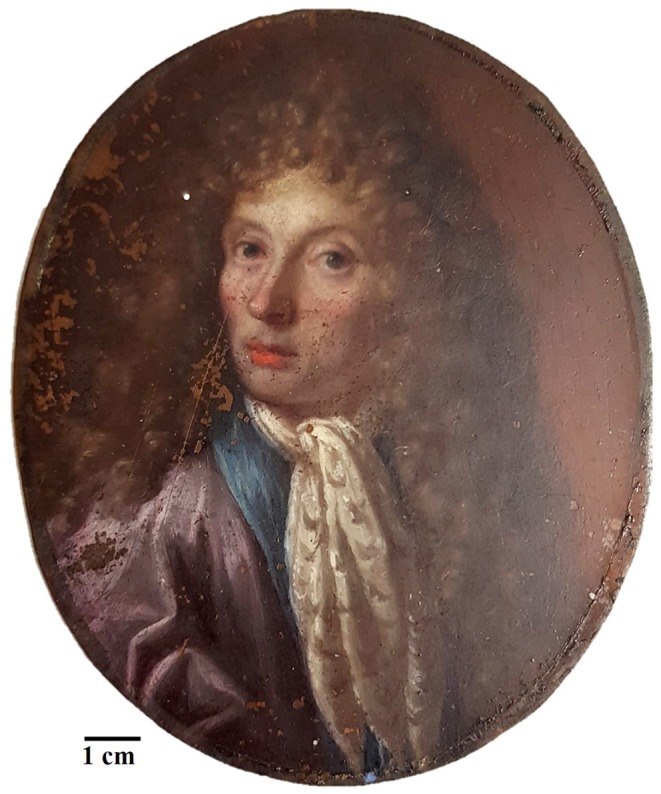
Picture of the painting on metal plate under investigation.

This work aims to propose a good analytical practice based on a multi-spectroscopic approach to characterize the microchemical and microstructural composition of these artworks and of their degradation products as it is done for painting on canvas (Miliani et al., 2010; Pitarch et al., 2014). This approach will allow to minimize and, in most cases, to make unnecessary the use of destructive analyses. A miniature portrait on a copper-based substrate (Figure 1) was analyzed by the means of multispectral imaging methods (UV, IR, and IRFC) (Cosentino, 2014), optical microscopy (OM), scanning electron microscopy coupled with energy-dispersive X ray spectrometry (SEM–EDS) (Haswell et al., 2006; Ingo et al., 2013), Fourier-transform infrared spectroscopy (FTIR) (Spring et al., 2005; Van der Weerd et al., 2005; Miliani et al., 2007) and macro X ray fluorescence (MA-XRF) (Cesareo et al., 2016; Iorio et al., 2019).

## Materials and Methods

### Artifact

The artwork under investigation belongs to a private collector and it is an oil painting representing the portrait of a nobleman painted on a metal plate as substrate (Figure 1). Unfortunately, it is not possible to attribute the painting to a specific artist as well as to a specific age, but due to the composition, the technique and the pigments used it is possible to presume that it was produced between the end of the 17th and the beginning of 18th century.

### Multispectral Imaging Methods

#### Ultraviolet-Visible Fluorescence Photography (UV)

Pictures of ultraviolet fluorescence of the painting were collected by a Canon EOS 500D camera equipped with 18–55 mm lens by illuminating the artifact with a Wood's lamp (E27, 160W).

#### Infrared (IR) and Infrared False Color Reflectography (IRFC)

Infrared pictures (IR) were acquired in the range 400–1,100 nm using a MICRO IR 20 camera charge-coupled device with a silicon detector CCD equipped with 12.5 mm lens (f 1.3) and a VIS and IR80 filters.

### Optical Microscopy (OM)

Investigations were performed by using Leica MZFLIII and Leica LAS multi-focus stereo microscopes equipped with a digital camera (Leica DFC 320).

### Field-Emission Scanning Electron Microscopy Coupled With Energy-Dispersive X Ray Spectrometry (FE-SEM–EDS)

The artifact was positioned inside the analytical chamber of the microscope without any surface preparation. The metal part of the artifact (verso) was connected with the microscopy stub using copper wires. For the investigation, a high-brilliance and high-spatial-resolution LEO Gemini 1530 (Zeiss, Germany) field emission scanning electron microscope (FE-SEM) was used. The instrument was equipped with an INCA 450 (Oxford Instruments Analytical, U.K.) energy-dispersive X-ray spectrometer (EDS) and four-sector back-scattered electron (BSE) detectors. Images were recorded at an acceleration voltage of 20 kV. The EDS was calibrated using RXS-36M commercial metals reference standard.

### Fourier Transform Infrared Spectroscopy (FTIR)

FTIR analyses were performed on the painting surface without any preparation using a Thermo Scientific Nicolet™Continuμm Infrared Microscope operating in reflectance mode. The FTIR microscope is equipped with an MCT-A (Mercury Cadmium Telluride) detector cooled by liquid nitrogen and a 15× Thermo—Electron Infinity Reflachromat objective with a tube factor of 10×. All spectra were acquired in the range 4,000–650 cm^−1^, at a spectral resolution of 4 cm^−1^. A total of 32 scans were recorded and the resulting interferograms averaged. Data collection and post-run processing were carried out using Omnic™ software.

### Macro X-Ray Fluorescence (MA-XRF)

MA-XRF analyses were performed by a scanner prototype built by the INFN Roma TRE division and Ars Mensurae (Iorio et al., 2019). The scanner has an exchangeable head attached to a motorized x-y stage. The head is made of a low-power Moxtek® tube with a Ta target and collimated to 1 mm, a silicon drift detector (SDD), and a twin-laser focusing system. The images resolution is about 1 mm^2^ per pixel. The tube operated at 37 kV and 17 μA with a dwell-time of 1 s. The XRF scanner was calibrated using SRM 1115—Commercial Bronze Standard for Optical Emission and X-ray Spectroscopic Analysis (NIST standard reference material) with the following composition expressed as % of mass fraction: Copper 87.96, Zinc 11.73, Lead 0.013, Iron 0.13, Tin 0.10, Nickel 0.074, and Phosphorus 0.05.

## Results and Discussion

### Metal Substrate

**Figure 2:**
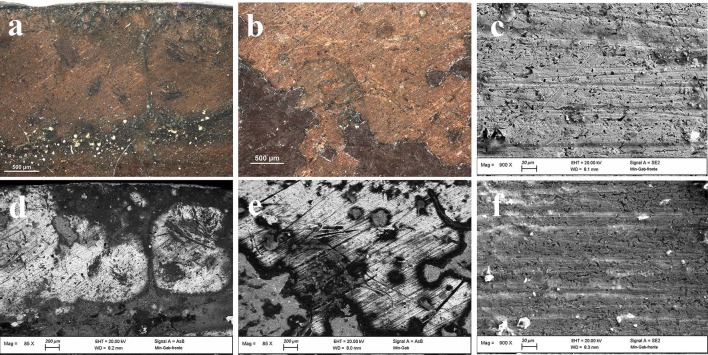
OM **(a,b)** and FE-SEM **(c–f)** images of painting failures showing the underlying metal plate with working marks.

**Figure 3:**
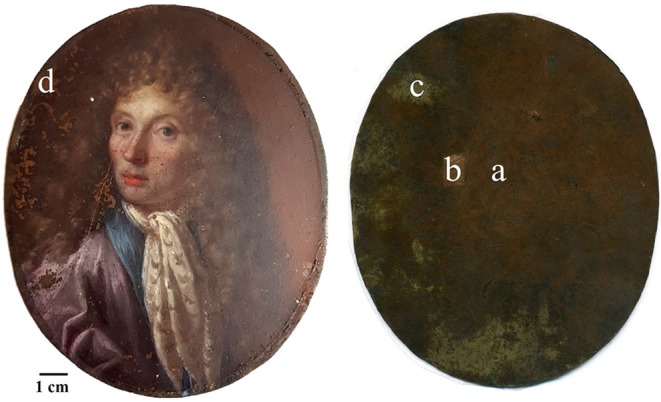
EDS points of analyses.

The metal plate used as substrate was investigated on both sides of the painting. The back side was easily reachable because of the absence of a painted layer. However, the accessibility of the front side was limited to those areas where painting failures exposed the metal surface underneath. Therefore, only few area of the front side were investigated. The first visual characterization of both sides was performed by optical microscopy. The main difference between the two sides is the presence of parallel lines engraved on the front of the plate (Figures 2a,b), that are not present on the back. In order to characterize these lines and due to its limited dimensions, the artwork was positioned inside an FE-SEM chamber and analytical measurements were performed directly on its surface. The FE-SEM images (Figures 2c–f) allowed to measure the width of this marks that can be grouped into 3 different ranges: a large one of 7.56 ± 0.45 μm, a medium one of 4.64 ± 0.35 μm and a small one of 2.82 ± 0.35 μm. Because of the consistency in width and direction, these lines seem to be intentional working marks made by the artist using a specific tool. Previous studies (Horovitz, 1986; Komanecky, 1990; Vega, 2016) suggested that, for this particular painting technique, artists used to prepare the metal surface in order to allow a better hold of the oil paint. The presence of these marks confirms these studies, revealing the intention of increase the surface roughness of the substrate to enable the adhesion of the paint layers to it. The two sides were also investigated by EDS in order to characterize the elemental composition of the metal plate. Three different area of the back side and one of the front side were analyzed (Figure 3 and Table 1).

**Table 1:** Elemental composition of the metal plate by EDS analyses.

**Analised area**	**C**	**O**	**AI**	**Si**	**S**	**Cl**	**K**	**Ca**	**Fe**	**Ni**	**Cu**	**As**	**Sn**	**Pb**
Back side—No Patina	12.80 ± 4.14	5.24 ± 1.13	0.05 ± 0.07	ND	0.57 ± 0.08	0.45 ± 0.05	ND	ND	0.15 ± 0.21	0.07 ± 0.10	77.29 ± 5.68	0.18 ± 0.14	3.09 ± 0.14	0.13 ± 0.18
Back side— White Patina	9.75 ± 0.88	11.05 ± 7.26	0.10 ± 0.13	0.11 ± 0.16	0.68 ± 0.68	0.60 ± 0.49	ND	ND	0.19 ± 0.26	ND	65.03 ± 9.11	ND	12.35 ± 1.22	ND
Back side—Red Patina	29.28 ± 0.17	15.32 ± 0.55	0.17 ± 0.01	0.38 ± 0.04	1.18 ± 0.08	0.96 ± 0.12	ND	0.15 ± 0.21	0.51 ± 0.01	0.05 ± 0.07	48.23 ± 0.54	0.05 ± 0.06	3.50 ± 0.29	0.44 ± 0.06
Front side	37.26 ± 8.38	11.34 ± 0.05	0.24 ± 0.03	0.58 ± 0.10	0.31 ± 0.04	0.29 ± 0.05	0.17 ± 0.06	ND	0.08 ± 0.11	ND	49.00 ± 7.98	0.05 ± 0.07	0.09 ± 0.12	0.62 ± 0.04

*Results are showed in wt%. ND, not detected*.

On the back, the analyses were performed on one area with the original red patina, one area with the bare metal exposed after a mechanical cleaning by scalpel, and also one area showing a whitish patina. In particular, analyses on the bare metal were performed in order to obtain an indication about the original chemical composition of the plate. Indeed, without cleaning, the original alloy signal could be attenuated by the presence of corrosion products on the metal surface resulting in the underestimation of minor elements. In Table 1, EDS results show that the metal substrate is made of a ternary bronze (Cu, Sn, Pb) with the presence of impurity such as Al, S, Cl, Fe, Ni, and As. The presence of these elements is quite common in ancient copper-based alloys and it is due to the smelting and uncompleted purification processes of copper from its ores (Craddock, 1976, 1977, 1978). The composition of the plate resulted to be similar on both patinated sides, with a higher contribution of those elements coming from the external environment and interacting with the metal. However, the content of tin appears to be significantly different. This result, paired with the composition of the whitish area on the back mainly containing tin corrosion products, may suggest a phenomenon of differential corrosion and surface enrichment in Sn at the back of the plate. Indeed, the back side was constantly exposed to the environment while the front was protected by the paint film that acted as a “coating.” Therefore, it is possible that the measured composition is the result of Sn concentration gradient that increases moving from the center toward the surface of the plate. Since the front side is partially protected from corrosion phenomena, the concentration of Sn has not increased there. To confirm this hypothesis, it would be interesting to analyze a cross-section of the metal substrate that would allow a better characterization of the chemical composition of the plate. Unfortunately, due to the general good conservation state of the painting, it was not possible to perform this supplementary investigation without jeopardizing the integrity of the artifact.

### Paint Layers

**Figure 4:**
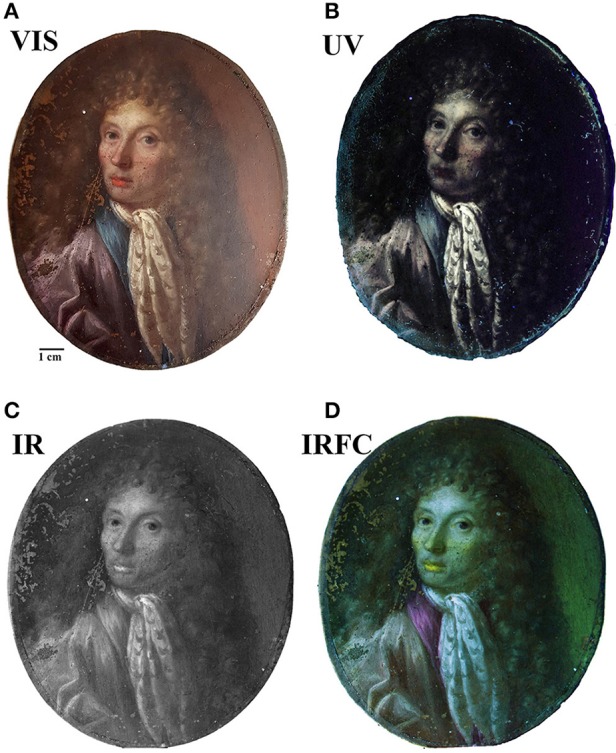
Painting images by imaging techniques: **(A)** visible; **(B)** UV-induced visible fluorescence; **(C)** IR Reflectography. **(D)** IR false-color images.

A first characterization of the painted layers was performed by multispectral imaging techniques as UV fluorescence, IR and IR false color (IRFC) photography on the entire surface of the painting (Figure 4). This allowed to determine the possible presence of an underlying preparatory layer as well as underdrawings, pentimenti or alterations. Also, these techniques can detect the presence of protective layers, retouches above and/or below the varnish and any other restoration intervention. Furthermore, it was possible to obtain preliminary informations about the pigments used in the upper layers (Cosentino, 2014). From the UV picture it is possible to observe, on the left edge, the presence of a blue fluorescence due to some residues from an old protective varnish. This evidence indicates an almost complete intervention of cleaning of the varnish that was most probably oxidized. The small dark spots with no fluorescence are due to non-retouched failures of the painting, while the areas on the face and on the neckcloth seem to be re-painted areas. This hypothesis is supported by the IR pictures results, where the different transparency of the pigments to the radiation showed the unevenness of the trait due to small retouches. Furthermore, the investigation by IR radiation did not show pentimenti or other modification to the composition as well as no underlying preparatory drawing. However, it is important to highlight here that the absence of a light-colored preparatory layer pose some limitations to the technique and does not allow to observe the high chromatic contrast that would results from a dark trait on a light background. Therefore, it is not possible to fully exclude the existence of a preparatory drawing even though no evidence of its presence were found. The final imaging technique applied to this painting was the IRFC photography, that allows to suggest the possible use of some pigments from its chromatic response. The red pigments from the lips, the skin, the eyes inner corner and part of the brown background resulted as a yellow tone which is usually due to the presence of cinnabar. Also, the red-purple resulting color of the collar, the eyes and partially of the blouse, might indicate the presence of lapis lazuli pigment. It is of uppermost importance to underline that, although IRFC results are reliable and of great importance for painting characterization as non-invasive technique, they only give an indication about the possible pigments used and cannot be substitute to chemical analyses. Therefore, to confirm IRFC results, the characterization of the pigment needs to be done through complementary chemicalbr analysis techniques.

**Figure 5:**
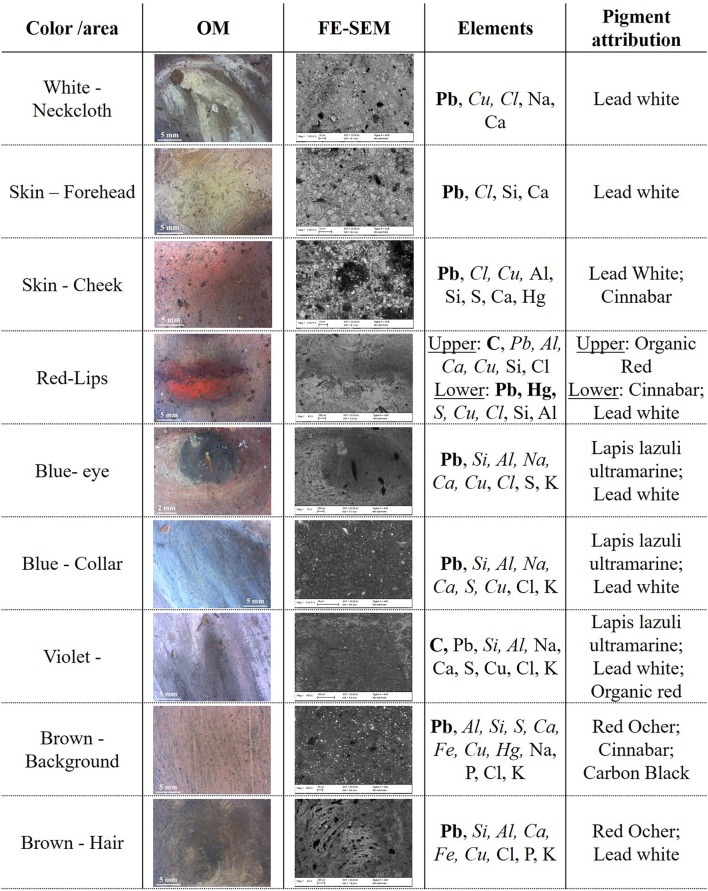
OM and FE-SEM pictures for each color shade of the painting. EDS results are showed in element wt% concentration range: higher than 10% (bold), between 10 and 1% (*italic*) and lower than 1% (regular). The C and O content is omitted, although always present, since their presence do not provide an indication for the pigment attribution. The pigment attribution is proposed based on the detected element.

In order to identify pigments and binder, EDS, MA-XRF and μFTIR were performed. In Figure 5 results from EDS analyses for each color are summarized. The white pigment used for the neckcloth and the skin contains mainly Pb suggesting the use of lead white [(PbCO_3_)_2_·Pb(OH)_2_]. Furthermore, a high content of Pb was found on the entire painting surface but mainly in the bright areas. This is due to its use in mixture with other pigments to brighten them and create a chiaroscuro effect in the composition. Concerning the skin color, in addition to Pb, the presence of S and Hg indicated the use of cinnabar or vermillion (HgS) in combination with lead white. Cinnabar and vermillion are red pigments that have the same chemical composition and differ only for their origin, being a natural mineral the first and an artificial synthetic product the latter. The two forms of this red pigments are not distinguishable using this analytical technique. The presence of cinnabar was also detected in the lower lip of the figure, while, in the upper lip, this pigment was not found. The disappearing of Hg and S corresponded to a relevant increase in the content of C suggesting the use of a red organic pigment that resulted in a darker red color. The blue pigment used for the eye iris and for the collar of the blouse contained Si, Al, Na, S, that indicate the use of lapis lazuli pigment (Na_8−10_ Al_6_Si_6_O_24_S_2−4_). This pigment was one of the most refined and expensive among all pigments and during the Renaissance it coasted even more than gold (Roy, 1993; Eastaugh et al., 2007). Therefore, the use of this pigment was exclusively devoted to refined and high-quality paintings. Lapis lazuli was also probably used to paint the blouse in mixture with the organic red pigment already detected in the upper lip in order to create the purple color. The brown background contains at the same time Fe, Al, and Ca that are the main component of brown ocher as well as a Hg and S, suggesting a mixture of the ocher with cinnabar, to enhance the red tone. The brown shade of the hair, on the contrary, was made using only the ocher. From this first chemical analysis, it is worth to notice that the indications given by the IRFC characterization were confirmed.

**Figure 6:**
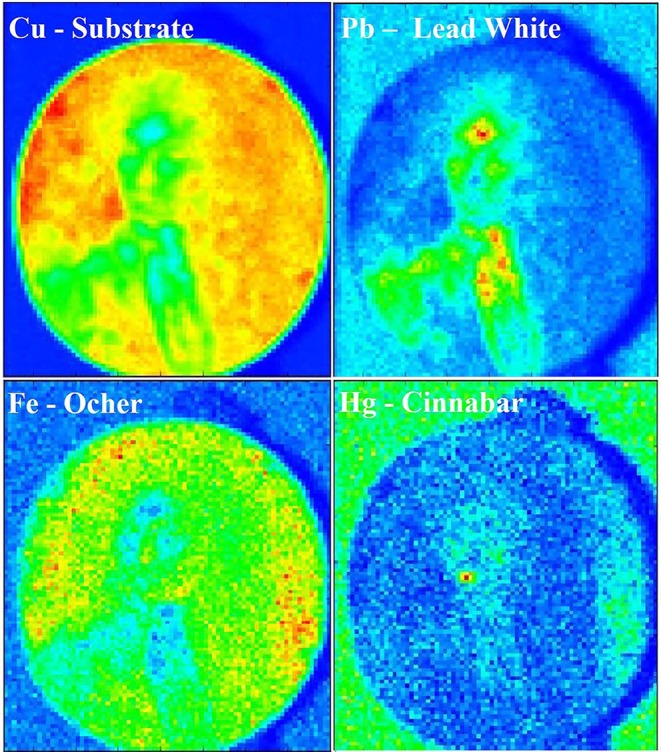
MA-XRF chemical map of the painting showing the distribution of Cu, Pb, Fe, and Hg. The intensity scale goes from blue (no detection of the element) to red (highest concentration of the element).

A combined chemical and visual characterization of the pigments was performed by MA-XRF, which resulted in the elemental distribution map of the painting. The main elements identified in each pigment can be used to map the white (Pb), the red (Hg), and the brown (Fe) color, as showed in Figure 6. However, due to the instrumental set-up, light elements as Na, Al, Si, and S that are present in the lapis lazuli could not be detected and, thus, this pigment was not mapped. In addition to the signals of the main pigments components, it is interesting to look at the Cu signal coming from the metal support, that can provide insights about both the thickness and the characteristics of the superposing pigments. In fact, the decrease of Cu signal corresponds to those areas where Pb is present and the higher is the Pb signal the lower is the Cu one. This is explained by the fact that Pb has shielding properties toward X-rays and therefore, the higher are its concentration and thickness the higher will be its shielding effect. In general terms, the use of this technique is comparable to EDS, giving the same analytical results, made exception for the lighter elements detection. However, EDS analyses cannot be performed on large objects, due to the limited analytical chamber dimensions. Furthermore, most of the time it is mandatory to perform *in-situ* measurements, making XRF the best techniques for such situations. Moreover, this technique combines the advantages of a single spot chemical analyses, as EDS or classic XRF, with the more straightforward visual interpretation given by imaging techniques as UV, IR, and IRFC. If needed, the use of this technique would allow to detect hidden paintings below the outermost surface, due to the difference in composition between pigments used in different time of history (Saverwyns et al., 2018). Therefore, even if time consuming, the application of MA-XRF for the study of this kind of artifact allows to have many information by a non-invasive approach and to have an easier discussion of the results between scientist and conservators or art-historians.

**Figure 7:**
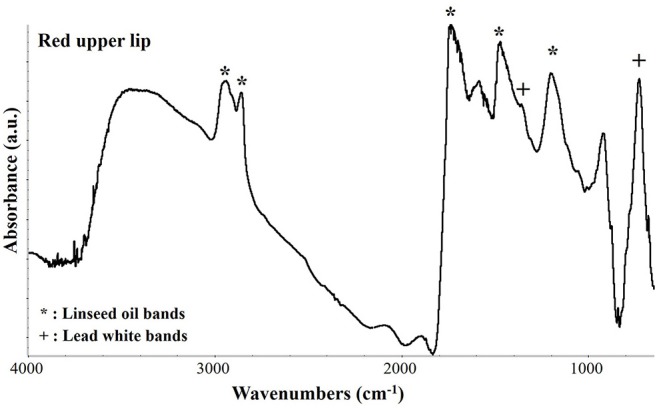
μFTIR absorbance spectrum collected on the upper lips in correspondence of a dark red pigment. The characteristic peaks of linseed oil and of lead white are indicated by an asterisk (*) and a cross (+), respectively.

The characterization of the organic materials of the painting, such as the organic medium and the red pigment of the upper lips, was performed by μFTIR analysis (Vetter and Schreiner, 2011). In Figure 7 the resulting spectrum acquired on the upper lips is shown. The peaks at 2,963, 2,937, 2,858, and 1,465 cm^−1^ are characteristic of C-H vibrational modes while the wide and asymmetric peak at 1,737 cm^−1^ indicates the C=O bond. All these peaks are characteristic of an aged linseed oil that was most probably used as binder of this painting. Regarding the red pigment, the FTIR spectra were not conclusive since the only other peaks present were those of lead white at 1,400, 1,358, and 7,60 cm^−1^. On the bases of all the investigation performed, it can be possible to presume that the red pigment could be a red lake. Indeed, because of the low content of dyestuff in traditional red lake pigments it would be very difficult to detect it in a mixture with lead white. In fact, the μFTIR spectrum would be easily dominated by the bands of the inorganic white pigment and of the binder.

**Figure 8:**
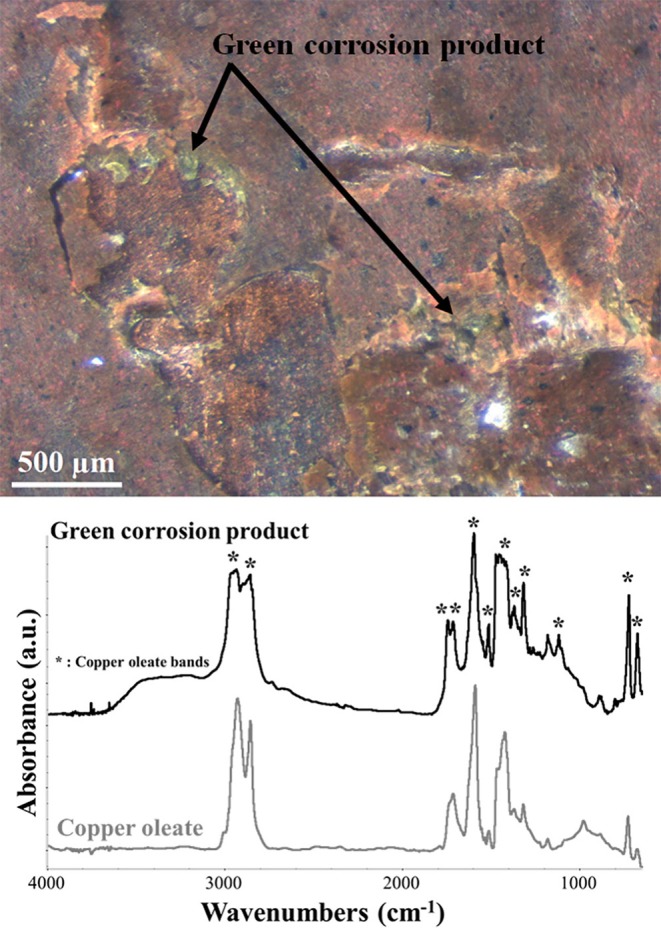
OM and μFTIR absorbance spectrum picture of the green degradation products found at the paint-metal interface. The spectrum of the product (black) is compared with the reference spectrum of copper oleate (gray). The asterisks indicate the corresponding copper oleate peaks in the green degradation product spectrum.

Finally, μFTIR spectroscopy was used to analyze some characteristic green corrosion products that were found at the interface between the paint layers and the copper plate (Figure 8). These products were only visible in those areas with painting failures and their presence resulted in a very brittle paint film. The corrosion product was identified as copper oleate, with its characteristic bands at 2,960, 2,930, 2,855, 1,592, 1,509, a broad band between 1,469, and 1,410, 1,316, 1,281, and 1,114 cm^−1^ (Robinet and Corbeil, 2003; Deng et al., 2014). The presence of metal soaps at the paint/copper interface is due to the interaction between the fatty acid of the binder and the metal ions of the substrate. Therefore, as suggested by other authors (Low et al., 1967; Pavlopoulou and Watkinson, 2006; Casadio et al., 2019), the formation of this corrosion product has to be expected in this kind of artifacts and possibly monitored in order to avoid brittleness of the paint film.

## Conclusions

In this work, a non-invasive analytical approach for the characterization of a painting on metal plate was proposed and performed. These kind of artworks present typical issues of composite artifacts made of two different materials. The use of a wide range of spectroscopic analytical techniques allowed to tackle both the paint film and the metal plate as well as new products originated from the metal/paint interaction. The micro-chemical and microstructural approach allowed to characterize the metal substrate, its composition and its preparation that the artist performed in order to obtain the best outcome for the painting. Furthermore, the full characterization of the painting with its pigments and binder, the preparatory layer and the conservation-restauration interventions was performed. Finally, it was possible to identify newly formed compounds resulting from the interaction between the metal plate and the painting binder. As future perspective, it would be interesting to further investigate a cross-section of the metal plate. This would allow to better characterize its composition as well as to look at the nano and microstructure of the metal bulk in order to obtain useful information about the working processes used to produce the plate. To conclude, the non-invasive multi-spectroscopic approach used on this artifact can be proposed as a good analytical practice to be adopted for the study of oil paintings on metal plates as it is already made for oil on canvas. The analyses allowed to obtain valuable information about the painting technique and the conservation state of the artwork that can be used by the conservator-restorers to propose the best conservation method and materials for the conservation-restoration on these artifacts.

## Data Availability Statement

The datasets generated for this study are available on request to the corresponding author.

## Author Contributions

MA designed the experiments and prepared the manuscript. CR and MP performed the FE-SEM characterization. SR carried out imaging and MAXRF characterization. MA, CG, and MPS performed FTIR characterization.

### Conflict of Interest

SR was employed by the company Ars Mensurae. The remaining authors declare that the research was conducted in the absence of any commercial or financial relationships that could be construed as a potential conflict of interest.
